# A Rare Presentation of Glucose-6-Phosphate Dehydrogenase Deficiency

**DOI:** 10.7759/cureus.63879

**Published:** 2024-07-05

**Authors:** Neha Tyagi, Varsha Premkumar, Manojkumar G Patil, Sampada Tambolkar, Shailaja V Mane

**Affiliations:** 1 Pediatrics, Dr. D. Y. Patil Medical College, Hospital and Research Centre, Pune, IND

**Keywords:** x-linked genetic diseases, jaundice, viral hepatitis a, acute hemolytic anemia, glucose-6-phosphate-dehydrogenase deficiency (g6pd)

## Abstract

Approximately 400 million individuals globally experience glucose-6-phosphate dehydrogenase (G6PD) insufficiency, an enzymatic condition that may be hazardous. Because of mutations in the *G6PD* gene, which result in functional variants alongside a variety of biochemical and clinical symptoms, this condition is an X-linked hereditary genetic disorder. Our case is that of a 12-year-old male child who presented with acute liver failure and later on, exhibited signs of hemolysis as well. We had to rule out the possibilities of acetaminophen toxicity and hepatitis A before reaching the conclusion that an underlying G6PD deficiency was being exacerbated by viral infection and simultaneous ingestion of non-steroidal anti-inflammatory drugs (NSAIDs).

## Introduction

Glucose-6-phosphate (G6PD) is typically present in all cells for the synthesis of nicotinamide adenine dinucleotide phosphate (NADPH). G6PD deficiency is an X-linked hereditary genetic defect due to mutations in the *G6PD* gene, which cause functional variants with many biochemical and clinical phenotypes. It is most commonly found in the Parsi community in Western India and within the Jhumli Thakur community of Rajasthan, India [1].

NADPH is the reducing agent for the production of deoxyribose, cholesterol, and fatty acids. It protects cells from oxidative stress concurrently. Hemolysis can result from oxidative damage to red blood cells [2]. Acute hemolytic anemia, neonatal jaundice, and chronic non-spherocytic hemolytic anemia are just a few of the symptoms of this illness, which predominantly affects red blood cells [3]. The three recognized trigger factors for symptomatic G6PD deficiency are (i) favism caused by fava beans, (ii) infections, and (iii) medications such as antimalarials, sulphonamides/sulphones, antibacterials, antipyretics, and analgesics.

Despite the fact that the majority of cases are clinically asymptomatic, the risk of developing symptoms persists. We describe one such case of a young male patient who presented with jaundice and hemolytic anemia, complicated by viral hepatitis. It was a long road to discovering the underlying G6PD deficiency, making recovery much more complicated.

## Case presentation

**Figure 1:**
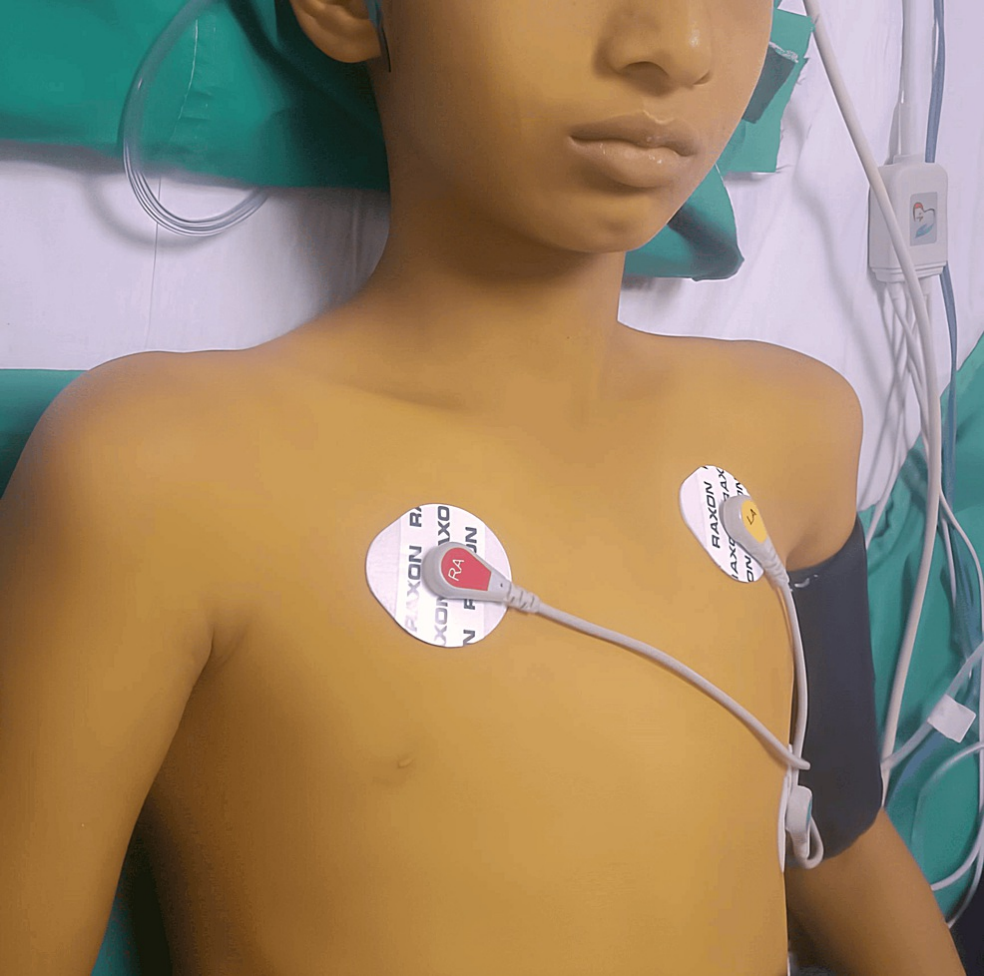
Yellow discoloration of the skin can be seen

**Figure 2:**
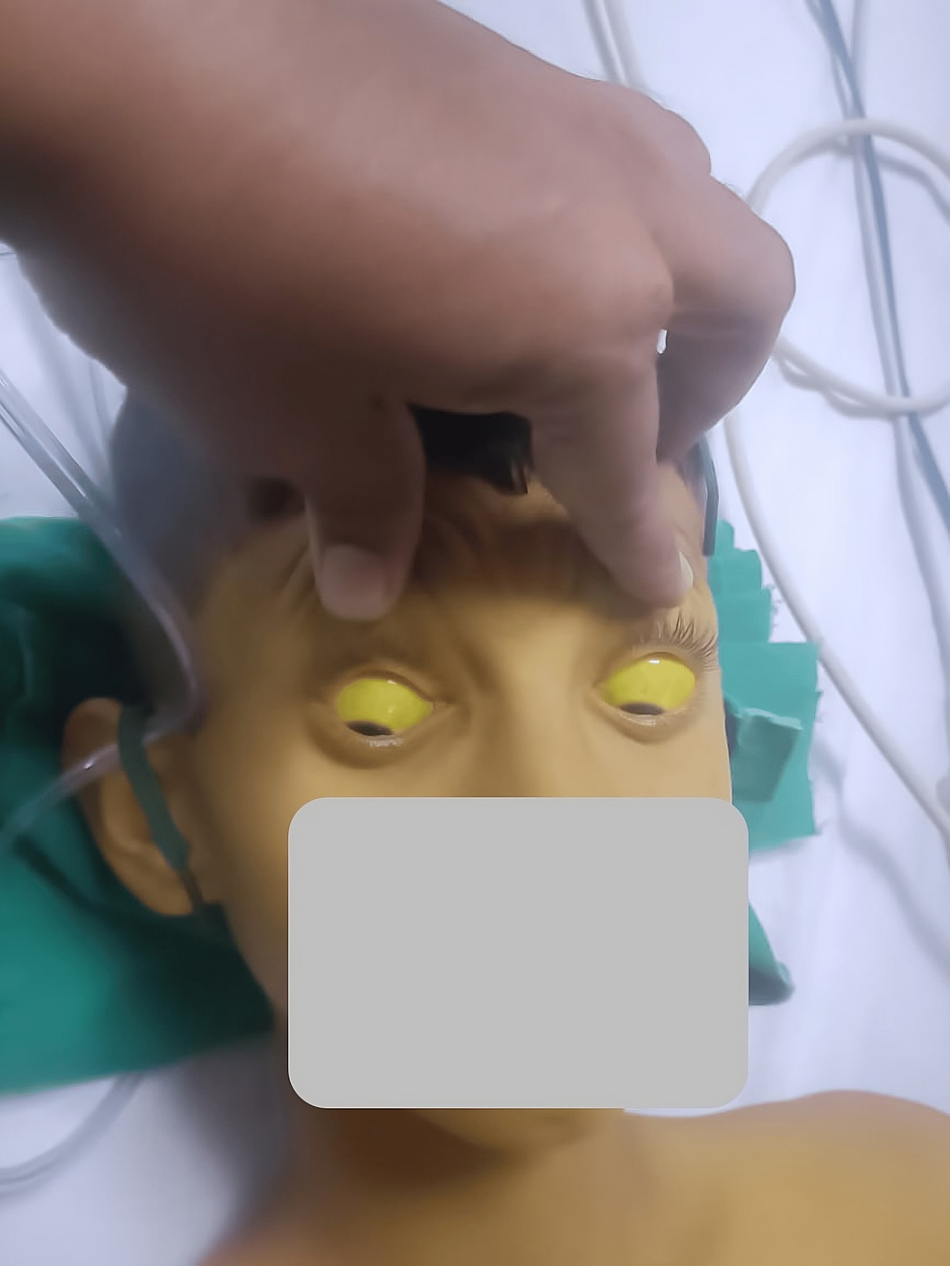
The icteric sclera

A 12-year-old male child belonging to the Pariyar community in Maharastra, India, born in the second birth order in a non-consanguineous marriage, was brought to our emergency department with complaints of vomiting and pain in the abdomen for four to five days. Pain was non-radiating and not associated with any altered bowel-bladder habits. In these four days, the mother also noticed progressively increasing yellow discoloration of the body, which started with the involvement of the sclera, followed by discoloration of the entire body, including the palms and soles. Figure 1 shows the yellow discoloration of the patient's skin, and Figure 2 shows the icterus in the sclera. 

**Figure 3:**
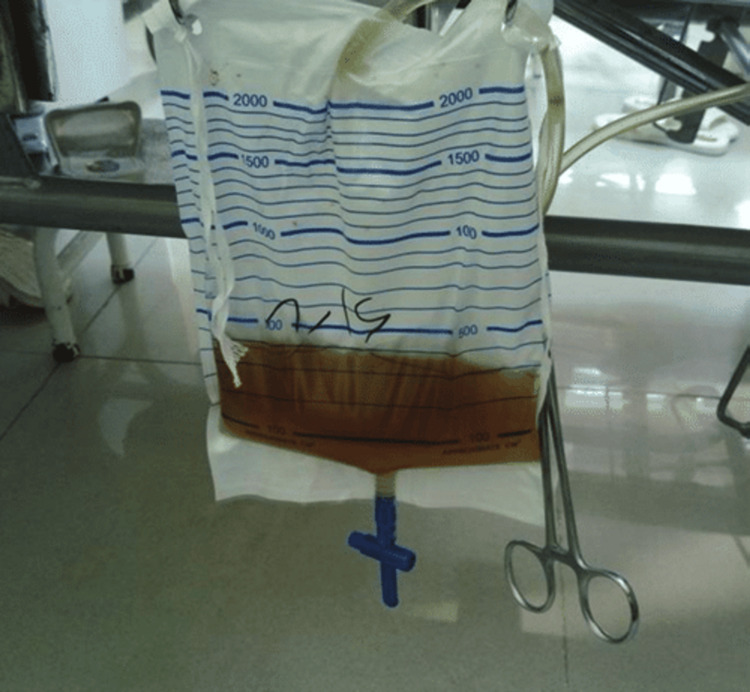
Dark-colored urine passed after admission

On further probing, the child gave a history of passing dark-colored urine (Figure 3), along with increased daytime sleepiness and disturbance of the sleep cycle experienced for three days.

On presentation, the child had two episodes of frank blood-tinged, loose stools, following which we admitted the child to the pediatric intensive care unit for further management. Further detailed history revealed a history of consumption of restaurant food one week ago, which was followed by two fever spikes for which over-the-counter non-steroidal anti-inflammatory drugs (NSAIDs) were also taken. There was no history of the intake of herbal medications or any long-term medications. There was no history of blood transfusions, hematemesis, melena or haematochezia, jaundice, or any liver disease in the family. The child had been born at full term via a lower segment cesarian section done in view of cephalopelvic disproportion, no resuscitation or neonatal intensive care unit admission was required, and there was no history of umbilical catheterization. The development of the child was appropriate for his age. He was scholastically at par with his classmates. He was immunized up to 18 months of age as per the national immunization schedule. The vitals and results of the general and systemic examination at the presentation are given in Tables 1-3.

**Table 1:** Vitals at admission

Variable	Values
Temperature	98.6^o^F
Heart Rate	63/minute
Respiratory Rate	20/minute
Blood Pressure	95/47 mmHg
Capillary Refill Time	Less than 2 seconds
Saturation	92% on room air (taken on oxygen by mask; saturation 100%)
Peripheral Pulses	Well felt

**Table 2:** General examination

Examination	Observation
Sclera	Icterus present
Lower palpebral conjunctiva	Pallor present
Palms and Soles	Icterus present
No Rash	
No signs of liver cell failure	

**Table 3:** Systemic examination

Examination	Observation
Per Abdomen	Soft, tender hepatomegaly 4 cm below the right costal margin, with liver span being 13 cm, left lobe not palpable, rounded edges, smooth surface. No splenomegaly. No ascites.
Cardiovascular	S1 S2 heart sounds normal, no murmurs
Respiratory	Bilateral air entry +, no added sounds
Neurological	Conscious, alert, no focal neurological deficits, Glasgow Coma Scale 15/15

The results of routine laboratory investigations for acute liver failure are given in Table 4. With respect to history and clinical findings, with a strong suspicion of acetaminophen toxicity, we started the child on an N-acetyl aminophenol infusion for 48 hours.

**Table 4:** Laboratory test results at admission

Test	Results	Reference Values
Hemoglobin (g/dl)	7.40	11-14.5
Total Leukocyte Count (/microL)	15900	4000-12000
Differential Leucocyte Count (N/L)	75/19	
Packed Cell Volume (%)	22.1	33-43
Urea	29	17-49
Creatinine (mg/dl)	0.31	0.19-0.49
Sodium (mmol/L)	131	138-145
Potassium (mmol/L)	4.93	3.50-5.10
Chloride (mmol/)	94	98-107
Total Proteins (g/dl)	6.40	6.4-8.3
Serum Albumin (g/dl)	4.50	3.5-5.2
Serum Ammonia (milimol/L)	30	20-120
Prothrombin Time (seconds)	14.9	10.24-12.71
Activated Partial Thromboplastin (seconds)	28.30	24.71-34.30
International Normalised Ratio	1.6	0.85-1.15
Total Bilirubin (mg/dl)	66.1	0.22-1.2
Conjugated Bilirubin (mg/dl)	43.4	Up to 0.5
Unconjugated Bilirubin (mg/dl)	22.7	0.1-1.0
Aspartate Aminotransferase (U/L)	1314	8-60
Alanine Transaminase (U/L)	2037	7-55
Alkaline phosphatase (U/L)	350	
Hepatitis A Virus IgG/IgM	Positive/Positive	-

Sonography screening of the abdomen and pelvis showed hepatomegaly of 14 cm with mildly hypoechoic liver parenchymal echotexture with a starry sky appearance. The portal vein measured 9 mm, and the spleen was normal in size.

The case was reviewed by the pediatric gastroenterologist, who suspected him to be in a Wilsonian crisis. As advised, labs sent serum ceruloplasmin, which was raised and could be explained by the involvement of the liver as urinary copper and Kayser Fleischer rings were negative. On further investigations, we noticed a trend of a fall in hemoglobin without any evidence of blood loss and an improvement in the international normalized ratio (INR) after starting IV vitamin K, as seen in Tables 5, 6.

**Table 5:** Falling hemoglobin suggestive of hemolysis

	Hospital Day 0	Hospital Day 4
Hemoglobin (g/dl)	7.40	6.8
Total Leukocyte Count	15900	5600
Differential Leucocyte Count (Neutrophil/Lymphocyte)	75/19	54/32
Packed Cell Volume (%)	22.1	20.8

**Table 6:** Trend showing normalising INR

	Hospital Day 0	Hospital Day 1	Hospital Day 2	Hospital Day 3	Hospital Day 4
Prothrombin Time (seconds)	14.9	11.4	17.7	19.0	15.7
Activated Partial Thromboplastin (seconds)	28.3	31.3	33.6	35.5	33.6
International Normalised Ratio	1.6	1.69	1.54	1.42	1.37

There were signs of hemolysis on the peripheral smear, for which further hemolysis workup was done. Retic count was 8%, corrected retic 3.9%. Lactate dehydrogenase was raised, 863 U/L; the direct Coombs test was negative but the indirect Coombs test was positive.

**Figure 4:**
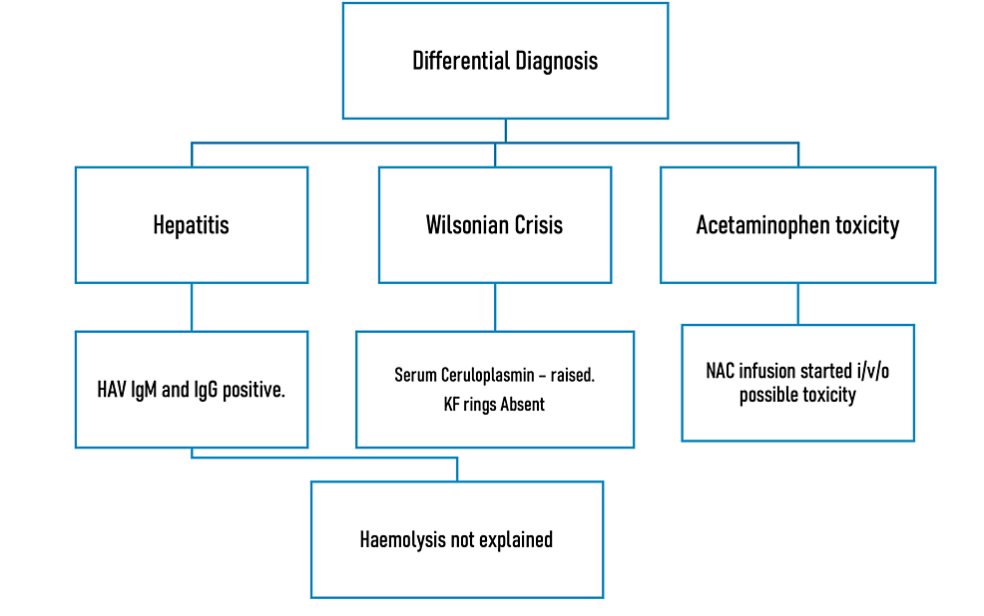
Differential diagnosis HAV: hepatitis A virus; KF: Kayser–Fleischer; NAC: N-acetyl cysteine

As per the history, examination, and investigations, we were able to narrow down our differentials to three: hepatitis, the Wilsonian crisis, and acetaminophen toxicity (Figure 4). However, none of these explained the hemolysis, and therefore we went ahead with further hemolytic workup. 

Haptoglobin was found to be less than 7.25 (normal: 30-200), a sign of intravascular hemolysis. Since our patient was a male child, it was a reason to strongly suspect an underlying G6PD deficiency in which the hemolysis was exacerbated due to infective etiology or the intake of NSAIDs. With this new differential in mind, we did a G6PD quantitative test by the decolorization method, which was positive, and the qualitative, kinetic method, which was also suggestive of deficient G6PD.

Treatment as per the liver failure protocol was already started. IV fluids dextrose 10% with concentrated ringer lactate (CRL), potassium chloride (KCl), and multiple vitamins injection, oral vitamins A, D, and E, IV vitamin K, IV cefotaxime, oral ursodeoxycholic acid, and N-acetylcysteine infusions were given during the initial 24 hours with suspicion of toxicity. Liver function tests and coagulation markers showed a falling trend as the treatment started to work on eliminating the infection, as shown in Table 7.

**Table 7:** Improving liver function tests

Liver Function Tests	Hospital Day 0	Hospital Day 1	Hospital Day 2	Hospital Day 3	Hospital Day 4	Hospital Day 6	Hospital Day 8	Hospital Day 10
Total Bilirubin (mg/dl)	66.1	59.6	47.9	30.2	17.19	10.8	9.7	7.9
Conjugated Bilirubin (mg/dl)	43.4	38.5	32	20.36	12.04	8.11	7.35	5.95
Unconjugated Bilirubin (mg/dl)	22.7	21.1	15.9	9.82	5.15	2.69	2.35	1.95
Aspartate Aminotransferase (U/L)	1314	740	367	142	84	69	72	98
Alanine Transaminase (U/L)	2037	1530	1252	817	559	333	234	152
Alkaline Phosphatase (U/L)	350	315	315	262	219	192	212	183

## Discussion

The frequency of G6PD insufficiency varies from 0% to 15% in various castes and ethnic and tribal groups in India, where it was first described more than 30 years ago [4]. The housekeeping enzyme, G6PD, is essential to every cell's ability to survive. In the first step in the hexose monophosphate (HMP) pathway, glucose-6-phosphate is converted to 6-phosphogluconolactone, and the cofactor nicotinamide-adenine dinucleotide phosphate (NADP) is reduced to NADPH. Although a G6PD deficit impacts all body cells, its main manifestation is hematopoiesis [5]. This is due to the fact that the HMP pathway is the sole source of NADPH in red blood cells, which is required to prevent oxidation of the cell and its hemoglobin. Red blood cells produce glutathione (GSH), which helps them recover from oxidative damage. The redox cycle of GSH is mediated by glutathione reductase and glutathione peroxidase, which are strongly linked to G6PD. Lower levels of G6PD make red blood cells more vulnerable to hemolysis in situations where oxidant drugs are administered, fava beans are consumed, or as a result of infection [6].

There are a few rare cases of G6PD deficiency being diagnosed in adolescents and at much older ages. However, it is easier to manage if diagnosed at birth during the newborn screening, preventing the life-threatening condition of hemolysis. In addition, reaching a diagnosis also becomes increasingly difficult with age as the probable differentials become vast.

In our case, there was no family history of any inherited blood disorder, nor was the caste of the family one of the vulnerable ones with a higher incidence of the disease. However, our major obstacle to reaching an accurate diagnosis was the presence of raised liver function tests but surprisingly normal serum proteins. The presence of a parallel viral infection was another factor misleading us. This made identifying the cause of the acute liver failure tricky until there were signs of hemolysis, which led us to investigate a wider spectrum of diseases.

Although acute hemolysis episodes can sometimes occur when oxidative stress levels are higher, patients with G6PD deficiency typically report chronic hemolysis. Transfusion-dependent hemolysis that resembles thalassemia major is known to occur in a small number of cases, and the severity of pallor and jaundice varies depending on the amount of hemolysis. G6PD deficiency is uncommon but treatable. It demands the careful avoidance of particular foods and medications that can cause a possibly fatal hemolytic event that calls for immediate medical attention and blood transfusions.

## Conclusions

In order to diagnose a patient and prevent unnecessary investigations and tedious referrals, history is crucial. Our patient was given a G6PD card mentioning a list of medications and food items to be avoided and the risks associated with their consumption. We asked the patient to show this card whenever visiting any doctor to avoid prescriptions for these medications. It is also to be kept in mind that neonatal screening could have helped us reach the child’s current diagnosis faster, with fewer diversions on the way. A latent or, therefore, asymptomatic deficiency of G6PD may clinically manifest under the appropriate conditions, even when evidence of past or current clinical evidence of G6PD deficiency is lacking. 
